# Automatic Fish Population Counting by Machine Vision and a Hybrid Deep Neural Network Model

**DOI:** 10.3390/ani10020364

**Published:** 2020-02-24

**Authors:** Song Zhang, Xinting Yang, Yizhong Wang, Zhenxi Zhao, Jintao Liu, Yang Liu, Chuanheng Sun, Chao Zhou

**Affiliations:** 1 College of Electronic Information and Automation, Tianjin University of Science and Technology, Tianjin 300222, China; 15620503230@163.com (S.Z.); yzwang@tust.edu.cn (Y.W.); 2 Beijing Research Center for Information Technology in Agriculture, Beijing 100097, China; yangxt@nercita.org.cn (X.Y.); zzx0525_2018@163.com (Z.Z.); jintaol@163.com (J.L.); liuyang951852682@163.com (Y.L.); sunch@nercita.org.cn (C.S.); 3 National Engineering Research Center for Information Technology in Agriculture, Beijing 100097, China; 4 National Engineering Laboratory for Agri-Product Quality Traceability, Beijing 100097, China

**Keywords:** aquaculture, automatic fish counting, hybrid neural network, machine vision

## Abstract

**Simple Summary:**

In aquaculture, the number of fish population can provide valuable input for the development of an intelligent production management system. Therefore, by using machine vision and a new hybrid deep neural network model, this paper proposes an automated fish population counting method to estimate the number of farmed Atlantic salmon. The experiment showed that the estimation accuracy can reach 95.06%, which can provide an essential reference for feeding and other breeding operations.

**Abstract:**

In intensive aquaculture, the number of fish in a shoal can provide valuable input for the development of intelligent production management systems. However, the traditional artificial sampling method is not only time consuming and laborious, but also may put pressure on the fish. To solve the above problems, this paper proposes an automatic fish counting method based on a hybrid neural network model to realize the real-time, accurate, objective, and lossless counting of fish population in far offshore salmon mariculture. A multi-column convolution neural network (MCNN) is used as the front end to capture the feature information of different receptive fields. Convolution kernels of different sizes are used to adapt to the changes in angle, shape, and size caused by the motion of fish. Simultaneously, a wider and deeper dilated convolution neural network (DCNN) is used as the back end to reduce the loss of spatial structure information during network transmission. Finally, a hybrid neural network model is constructed. The experimental results show that the counting accuracy of the proposed hybrid neural network model is up to 95.06%, and the Pearson correlation coefficient between the estimation and the ground truth is 0.99. Compared with CNN- and MCNN-based methods, the accuracy and other evaluation indices are also improved. Therefore, the proposed method can provide an essential reference for feeding and other breeding operations.

## 1. Introduction

In intensive aquaculture, the reliable estimation of fish biomass is essential for the aquaculture industry [1]. As a common biomass information of fish, the regular acquisition of the number of fish can help optimize the feeding process, control the breeding density, determine the optimal harvest time, and provide valuable input for the development of an intelligent production management system [2].

However, traditional fish counting mainly depends on manual sampling and direct counting. It is not only time consuming and laborious, but also a destructive contact method, which affects fish welfare and health status. The recently developed machine vision-based nondestructive testing method avoids damaging the water environment, thereby not affecting the normal behavior of fish, and is increasingly of interest for aquaculture, marine resources, and other research fields [3,4,5]. In addition, in far offshore mariculture, the water quality is good and the visibility of water is high. Moreover, the cost of the machine vision method is low, and the practicability is stablished, thereby providing a feasible scheme for fish state detection in aquaculture [6]. However, the underwater environment is restricted by the light conditions and noise; thus, it is difficult to distinguish the fish from the background. In addition, fish are free to move in the water, resulting in different shapes and serious occlusion problems. It is very challenging to realize fish counting underwater [1].

Predecessors have studied many fish counting methods based on machine vision. The general method is to use a machine learning method to realize fish counting after extracting fish image features. For example, information of the blobs was used to count fish fry [7] but the size of fry needs to be kept basically the same. Similarly, the area information of the outline was used to count fish [8], but the water level must be kept shallow to avoid overlapping. By extracting seven shape features, the least square support vector machine (LSSVM) achieves 98.73% accuracy for fish fry counting [9]. After using the Canny edge detection algorithm [10] to detect the outline of the fish shoal, blob detection realizes the fish counting [11,12]. A new algorithm based on endpoints of the skeleton was proposed to count the fish fry [13], which could overcome the fish overlap. The underwater environment is more complicated and the overlap is more serious, this method may not be accurate [1]. Recently, a fish counting method, including segmentation, contour detection, blob detection, and Kalman filter technology, has achieved an average accuracy of 97.47% [14]. In summarizing, when using the traditional machine learning method in image processing, sophisticated features must be extracted manually. To some extent, the performance often depends on the experience of experts. Despite the advantages of the traditional machine learning technology in addressing big complex data, its inherent effectiveness and scalability are not sufficient [15]. The underwater environment is complex, with stronger interference and noise. When the distinction between the fish and the background is not apparent and the fish are occluded from each other, it is difficult for traditional machine learning to realize fish counting. In reference [7], with the increasing number of fish, occlusion increases, and the estimation accuracy decreases. Compared with traditional machine learning methods, deep learning does not require sophisticated feature extraction engineering, which has strong adaptability and is easy to transform. The basic ideas and technologies of deep learning used in different fields are often transferrable [16,17]. In the era of big visual data in underwater observation, deep learning represents a practical solution.

Deep learning has shown advanced advantages in the field of animal computing, such as animal behavior analysis [18], animal recognition, and species classification [19,20], etc. In aquaculture, convolutional neural networks (CNNs) have gradually become the mainstream research model. For example, they have been used for fish behavior analysis [21], fish species identification [22], intelligent feeding [23], etc. Salman et al. [24] proposed a unified approach to detect freely moving fish in unconstrained underwater environments using a region-based convolutional neural network (R-CNN), which achieved 80.02% accuracy on the LifeCLEF 2015 fish dataset. Rauf et al. [22] proposed a deep CNN with 32 layers to identify fish species, thereby achieving the best performance on self-built dataset. The multi-layer convolution operation can automatically extract image feature information, including texture, shape, and position. According to the scene requirements, the final desired model is obtained through continuous training of the difference (loss function) between the predicted value and the ground truth. However, the receptive field of the shallow CNN is small, and only certain local feature information can be learned [25]. The deep CNN utilizes larger receptive fields and can learn more global information. It plays a greater role in scenarios where context information needs to be considered [26]. In addition to increasing the depth of the network and the size of the convolution kernel, the multi-column convolutional neural network (MCNN) [27] and the dilated CNN can also be used to increase the size of the receptive field. The MCNN uses CNNs with different kernel sizes to capture the feature information of different receptive fields, and the feature information of each CNN is ultimately merged into the output layer. The dilated CNN increases the receptive field by adding holes to the standard CNN [28].

To solve the above problems, this paper proposes a hybrid neural network model based on a multi-column CNN and a dilated CNN. Our network can realize the real-time, accurate, objective, lossless fish counting in far offshore Atlantic salmon mariculture. The structure of this paper is organized as follows: the first chapter is the introduction. The second chapter mainly introduces the construction of the datasets, basic theoretical methods, and proposed models. In the third chapter, we give the results of fish counting and discuss the performance of the proposed model. The fourth chapter is the conclusion.

## 2. Materials and Methods

### 2.1. Experimental Materials

**Figure 1:**
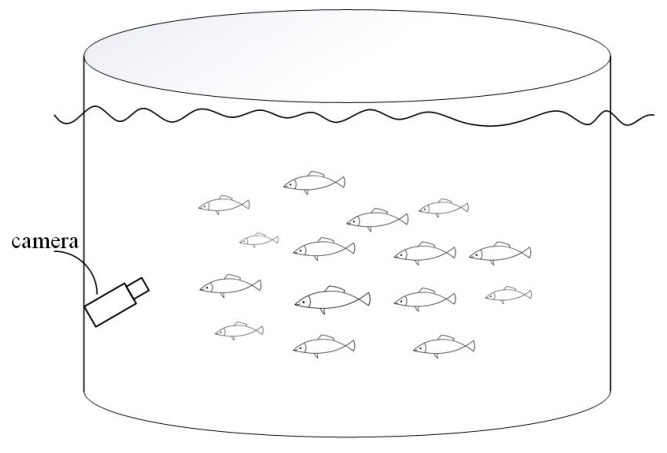
Data acquisition diagram.

Experimental video data were collected from the “Deep Blue No. 1” far offshore mariculture net cage located in the Yellow Sea of China, and were provided by Wanzefeng Fishery Co., Ltd., Rizhao, China. The fish farmed in the cages were adult Atlantic salmon, and videos of Atlantic salmon were collected underwater. The collection camera takes pictures of fish from the bottom up and forms a certain angle with the water surface to avoid the influence of vertical light on the acquisition. Figure 1 shows the collection diagram. The captured video has a resolution of 1920 × 1080 and a frame rate of 60 fps. Sequence images are extracted from the video data, frame by frame. In this experiment, no fish was harmed by stress, and the collection was conducted under conditions that did not affect its normal growth. The experiment did not involve animal ethical issues.

### 2.2. Dataset

#### 2.2.1. Data Preprocessing and Enhancement

The quality of the underwater image is reduced because of the influence of light and turbidity. For easy labeling, images need to be pre-processed and enhanced.

Due to the absorption and scattering of light as it propagates through water, underwater images often suffer from color shifts and reduced contrast. Therefore, to obtain better results, we use color correction and contrast enhancement [29] to improve the underwater images’ quality so that the images can be easily labeled. Inspired by the grey world hypothesis [30], a color correction strategy based on linear transformation is adopted. In 8-bit images, the pixels of the images are stretched to an average of 128 using a piecewise linear transformation. We define *S* as the input image and calculate the average, maximum, and minimum of the three RGB components. The basic form is as follows:

(1) $$S_{CR}^{c}=\{\begin{array}{c} \left(S^{c}-S_{mean}^{c}\right)\frac{S_{min}^{c}-128}{S_{min}^{c}-S_{mean}^{c}}+128,\;S_{mean}^{c}\leq 128\\ \left(S^{c}-S_{mean}^{c}\right)\frac{S_{max}^{c}-128}{S_{max}^{c}-S_{mean}^{c}}+128,\;S_{mean}^{c}>128 \end{array},$$

where c∈{R,G,B}, $S_{mean}^{c}$, $S_{max}^{c}$, and $S_{min}^{c}$ are the mean, maximum, and minimum in the c channel, respectively, and $S_{CR}^{c}$ is the corrected image. The average is used as the direction of the stretch. Due to the long wavelength of red light, it is easily absorbed in water, resulting in weak red component. Therefore, the formula needs to be fine-tuned to prevent overcorrection:

(2) $$S_{CR}^{c}=\{\begin{array}{c} S^{c}-\lambda \left(S_{mean}^{c}-128\right),\;P^{c}>0.7\\ \left(S^{c}-S_{mean}^{c}\right)\frac{S_{min}^{c}-128}{S_{min}^{c}-S_{mean}^{c}}+128,\;S_{mean}^{c}\leq 128\\ \left(S^{c}-S_{mean}^{c}\right)\frac{S_{max}^{c}-128}{S_{max}^{c}-S_{mean}^{c}}+128,\;S_{mean}^{c}>128 \end{array},$$

where λ is a positive parameter controlling the shift range [29], $P^{c}$ is a pixel value probability less than or equal to 40, and $S_{CR}^{c}=\min\left(\max\left(S_{CR}^{c},0\right),255\right)$ is used to avoid exceeding the pixel range.

**Figure 2:**
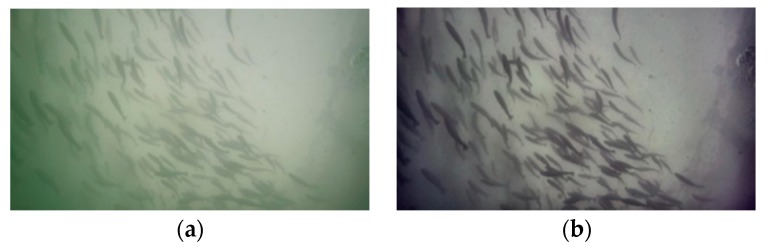
Image contrast before and after enhancement: (**a**) original image and (**b**) enhanced image.

After color correction, the underwater image is still blurry [29]; thus, it is necessary to enhance the contrast to highlight the objects and details. The basic idea is to find an appropriate modified image between the original image $S_{0}$ and the reference image $S_{r}$. Because they both contain different useful information, the goal is to find a balance between them. The form is as follows:

(3) $$F\left(E\right)=\alpha {\left|\left|E-S_{0}\right|\right|}_{W^{1,2}}^{2}+\left(1-\alpha \right){\left|\left|E-S_{r}\right|\right|}_{W^{1,2}}^{2},$$

where ${\left|\left|u\right|\right|}_{W^{1,2}}^{2}=\sqrt{{\left|\left|u\right|\right|}_{2}^{2}+{\left|\left|Du\right|\right|}_{2}^{2}}$ is the $W^{1,2}$ norm in the Sobolev space, *E* is the picture after color correction, α∈[0,1] is a positive parameter, and *D* denotes the difference operators. The result of image enhancement is shown in Figure 2, where Figure 2a is the original image and Figure 2b is the corresponding image after enhancement. The modified image improves the performance.

#### 2.2.2. Dataset Production

**Figure 3:**
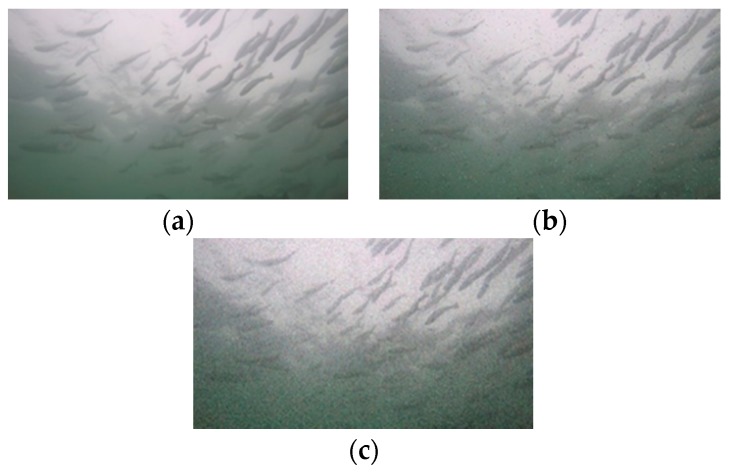
Original image and noisy images: (**a**) original image, (**b**) salt-and-pepper noise image, and (**c**) Gaussian noise image.

After image enhancement, 1501 original images were selected as the dataset. The original images are 1920 × 1080. In reducing the network input, the images are uniformly converted to 1280 × 720 pixels. Moreover, Gaussian noise and salt-and-pepper noise (as shown in Figure 3) with a variance of 0.001 are added to the original images to expand the dataset and increase the robustness of the network model [31].

**Figure 4:**
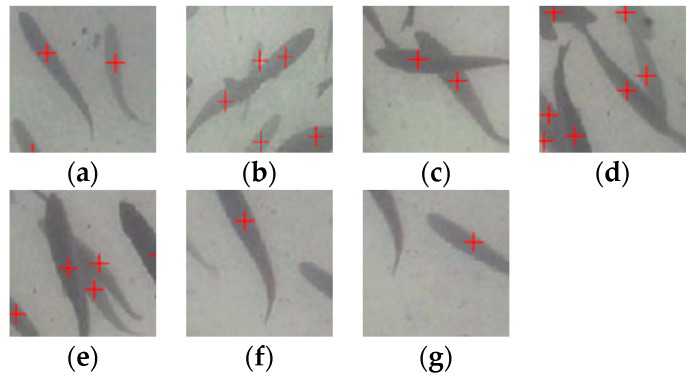
Examples of image annotation. (**a**) no overlap; (**b**) two fish overlap into a line; (**c**) two fish cross like “X”; (**d**) two fish cross like “V”; (**e**) three fish cross; (**f**) about half of the fish body appears; (**g**) a small part of the fish body appears.

The final dataset includes 6004 frames of original images, enhanced images, Gaussian noise images, and salt-and-pepper noise images. It is challenging to determine each fish’s position and the number of a fish shoal by labelling the same part of the fish. Therefore, the center of the fish is labelled (Figure 4a) in this study. For partially occluded fish, the center position of the largest exposed part is labelled as far as possible (Figure 4b–e). Approximately half of the fish body appears in the image (Figure 4f), while a small part of the fish body appearing in the image is not labelled (Figure 4g). Each label records the position of the corresponding fish, and the number of labels corresponding to the image indicates the number of fish in the shoal; thus, each label is a sample of the dataset.

**Figure 5:**
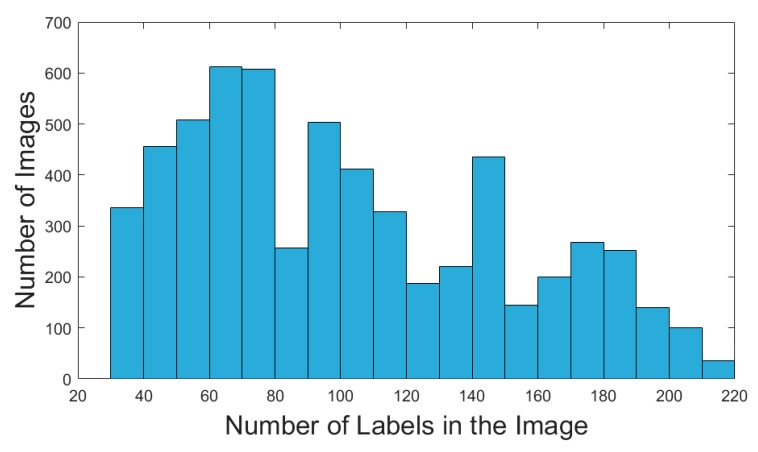
Histogram of the number of fish in the shoal of our dataset.

One doctoral student and three postgraduate students labelled 153,513 fish in total for approximately two weeks. The dataset contains a total of 614,052 labeled fish for which we have enhanced the dataset. MATLAB 2016a was used to generate the dataset of fish counting. Figure 5 shows the histogram of the number of fish in the shoal in the dataset. The maximum number of fish in the shoal is 214, the minimum number of fish in the shoal is 30, and the average number of fish in the shoal is 102.3.

### 2.3. Fish Counting based on a Hybrid Neural Network Model

#### 2.3.1. Fish Shoal Density Map

Inspired by crowd counting, we follow the method of crowd density estimation to achieve fish counting. Detection [32] and regression [33] are the two main methods used for crowd counting. Methods based on detection use a sliding window to detect objects one by one. This method has difficulty detecting partially occluded objects, and its performance is poor in crowded scenes. Regression methods calculate the number of specific objectives by learning the relationships among image features, which solves the problem of counting in large-scale crowded scenes. However, regression ignores the significance features, leading to inaccurate prediction in local areas. The density map [34] overcomes the shortcomings of the above two methods. It retains the local feature information and can obtain more fish distribution information while ensuring an accurate estimation.

The fish density map provides information about the two-dimensional spatial distribution of the fish shoal such as the position and number of fish in the region of interest in a frame for a specific time. Combined with continuous video sequences, it is also possible to calculate the speed information of the fish shoal movement. The density map shows the distribution of fish shoal in the image. When there are many fish in a small area, an abnormal situation has occurred. Its mathematical representation is as follows:

In a labelled image, if there is a label at pixel $x_{i}$, it is represented by a delta function $\delta \left(x-x_{i}\right)$. Hence, an image with *N* labels can be described by the function:

(4) $$H\left(x\right)=\sum_{1}^{N}\delta \left(x-x_{i}\right).$$

**Figure 6:**
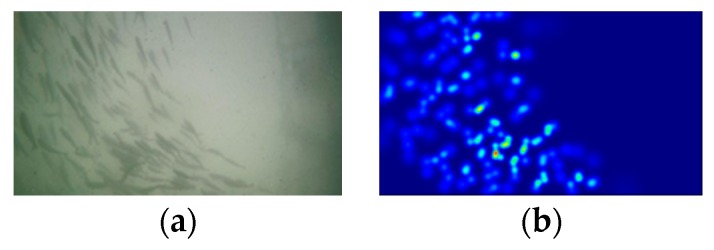
Diagram of density map: (**a**) original image and (**b**) corresponding density map.

Then, H(x) is convoluted with the Gaussian kernel $G_{\sigma }$, obtaining the continuous function $F\left(x\right)=H\left(x\right)*G_{\sigma }\left(x\right)$. However, this density function assumes that$\;x_{i}$ is an independent sample on the image plane. The fish images are all obtained from an underwater 3D scene, which suffers from perspective distortion. Different samples $x_{i}$ correspond to different-sized regions. To accurately estimate the number of fish, the distance between each label and the surrounding labels needs to be considered. The adaptive Gaussian convolution kernel $G_{\sigma_{i}}\left(x\right)$ is used for the convolution:

(5) $$F\left(x\right)=H\left(x\right)*G_{\sigma_{i}}\left(x\right),\;with\;\sigma_{i}=\beta {\bar{d}}_{i},$$

where ${\bar{d}}_{i}$ is the average distance between the label $x_{i}$ and its nearest *k* labels, and β=0.3 is an adjustable parameter based on reference [27]. The estimated density map generated by the model needs to be compared with the ground truth. The resulting error loss is propagated back to the network so that the training can be carried out in the direction of decreasing loss. The accuracy of the label directly affects the quality of the model training. Figure 6 shows the density map generated using adaptive Gaussian convolution kernel.

#### 2.3.2. Design of the Hybrid Neural Network

In this paper, a hybrid neural network model is proposed based on a multi-column CNN and a DCNN. The inputs of the network are images with labels (labels only for training), the corresponding outputs are the density map and the number of fish which is calculated by integrating (mathematically) the density map. The basic idea is to use a multi-column CNN in the front end to capture the feature information of different-sized receptive fields and use different-sized convolution kernels to adapt to the angle, shape, and size changes caused by the fish movement. Simultaneously, to reduce the loss of spatial structure information during network transmission, a wider and deeper DCNN is used in the back end.

##### Multi-Column Convolution Neural Network

The CNN has the characteristics of local perception and weight sharing. Local perception means that each neuron only perceives the local pixels of the image; this local information is merged at higher levels to obtain all the feature information of the image. Weight sharing reduces the complexity of the network model and the number of parameters. The CNN uses the original image as the input; it can effectively learn the corresponding features from a large number of samples and avoids a complex feature extraction process. However, convolution kernels with the same-sized receptive field are not sufficient to capture the characteristics information of fish with different sizes. The size of the convolution kernel is different in each column of the multi-column CNN; thus, the size of the receptive field is different to adapt to the change in fish size. Therefore, the front end network of this paper is based on the multi-column CNN to learn the feature information under different receptive fields.

##### Dilated Convolution Neural Network

Pooling layers (average pooling and maximum pooling) are widely used in neural networks. They are mainly used to reduce the dimensionality, compress data, reduce the number of parameters, control overfitting, and improve the fault tolerance of the model while maintaining the main features. However, with the deepening of the network and the stacking of the pooling layer, the image resolution continues to decrease, resulting in a loss of spatial structure information. The loss of spatial structure information may limit the accuracy of the network model and affect the migration of the model to other tasks. Once this type of detail information is lost, it is almost impossible to recover it through upsampling and training. In certain complex scenes, it is necessary to consider the spatial structure information [35].

Dilated convolution retains more spatial structure information by increasing the receptive field. It achieves better performance in addressing imagery that needs global information or speech text that needs long sequence information such as for semantic segmentation [28,36], image super division reconstruction [37], object detection and classification [38]. The receptive field is enlarged without increasing the number of parameters and calculations. In dilated convolution, a small convolution kernel size *k* × *k* is increased to ${\left(kr-r+1\right)}^{2}$, where the dilation rate is *r*, which enables the flexible aggregation of multi-scale information and maintains the same resolution. 

**Figure 7:**
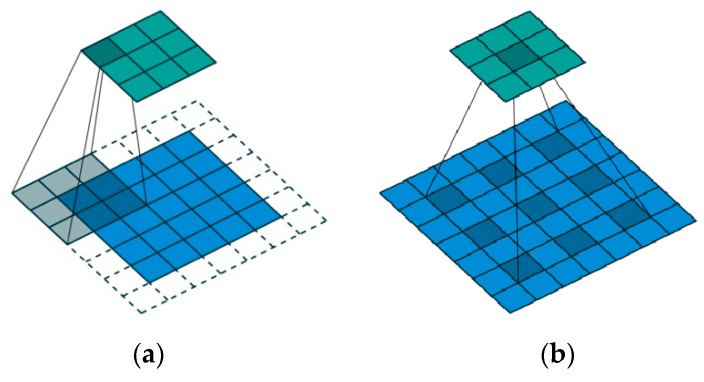
3 ×3 convolutional kernels with dilation rates of 1 and 2 [39]: (**a**) dilated convolution with dilation rate r = 1 and (**b**) dilated convolution with dilation rate r =2.

Figure 7a corresponds to a dilated convolution with kernel size 3 × 3 and dilation rate r = 1, which is the same as the standard convolution. Figure 7b corresponds to the dilated convolution with kernel size 3 × 3 and dilation rate r = 2; however, the actual convolution kernel size is still 3 × 3. For the 5 × 5 receptive field, only 9 points are convoluted with the 3 × 3 kernel, and the remaining points are skipped. The size of the receptive field is 5 × 5; however, only 9 points have non-zero weights, and the remaining points are zero. Although the convolution kernel is only 3 × 3, the receptive field has been expanded to 5 × 5. The dilated convolution expands the perception range without losing more information. Thus, the output of each convolution contains a more extensive range of information.

##### Design of the Hybrid Neural Network

**Figure 8:**
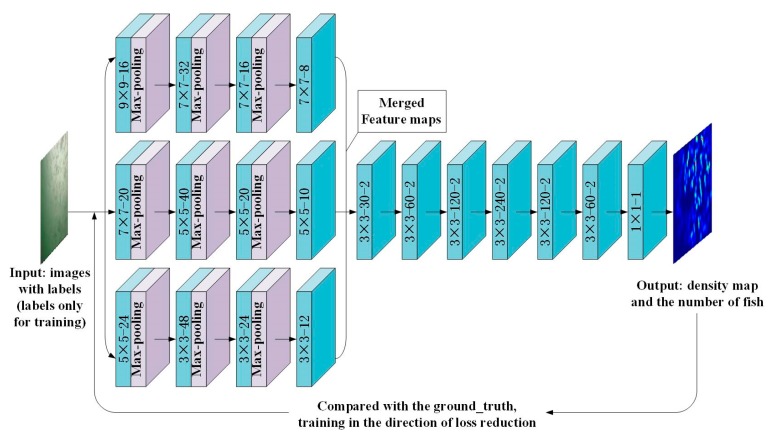
The structure of the proposed model. The convolutional layer’ parameters are denoted as “(kernel size)-(number of filters)-(dilation rate)”; max-pooling layers are conducted over a 2 × 2 pixel window with stride 2.

In this study, the front end of the proposed model is based on the MCNN [27], therein using a multi-column CNN to capture the feature information of different-sized receptive fields. Each column of the CNN uses a convolution kernel of different size to adapt to the changes in the fish body size and individual differences caused by fish swarm movement. The front end branches use three pooling operations. The back end uses dilated convolution instead of a pooling-convolution structure to reduce the loss of spatial structure information. Moreover, we deepen the network to mine more in-depth information. When the object is the same size as the receptive field, it is better to use more convolution layers and smaller convolution kernels [40]. Therefore, the convolution kernels of the back end are set to 3 × 3 and the dilation rate is 2. Considering a single underwater scene, to reduce the parameters of the network model, the network is not set as wide as visual geometry group-16 (VGG-16). In the last layer, the convolution layer is used instead of the fully connected layer such that the input image can be any size. Figure 8 shows the structure of the proposed model. The inputs are images with the labels (labels only for training), the final outputs are the corresponding density map and the number of fish.

### 2.4. Model Performance Evaluation Metric

In this study, the mean absolute error (MAE), root mean square error (RMSE), mean absolute percentage error (MAPE), and accuracy were used to measure the performance of the proposed model. MAE is one of the most basic evaluation metric and reflects the accuracy of the estimation. RMSE is more sensitive to extremum, and the large errors in the training process can impact the RMSE, which can be used to test the stability of the model. MAE and RMSE are greatly affected by the number of fish in an image. When both ground truth and the predicted value are small, even if the error ratio is large, the MAE and RMSE may be small; thus, it is difficult to correctly determine the performance of the model. The MAPE considers not only the error between the predicted value and the ground truth, but also the ratio between the error and the ground truth. The MAPE evaluates the model performance more comprehensively. The accuracy indicates the model performance directly in simplified terms. The formulas are as follows:

(6) $$MAE=\frac{1}{N}\sum_{1}^{N}\left|z_{i}-z_{i}^{GT}\right|,$$

(7) $$RMSE=\sqrt{\frac{1}{N}\sum_{1}^{N}{\left(z_{i}-z_{i}^{GT}\right)}^{2}},$$

(8) $$MAPE=\sum_{1}^{N}\left|\frac{z_{i}-z_{i}^{GT}}{z_{i}^{GT}}\right|\times \frac{100}{N},$$

(9) $$Accuracy=\left(1-\frac{1}{N}\sum_{1}^{N}\left|\frac{z_{i}-z_{i}^{GT}}{z_{i}^{GT}}\right|\right)\times 100\%,$$

where N is the number of test images, $z_{i}^{GT}$ is the number of fish in image i, and $z_{i}$ is the estimated number of fish in picture i.

## 3. Results and Discussions

In this experiment, a deep learning server was used for training. The hardware configuration includes two Intel(R) Xeon E5-2620 v3 CPUs @ 2.50 GHz, 48GB of memory (DDR4 2133MHz), 240GB solid-state drive, and NVIDIA GeForce RTX 2080Ti GPU with 11 GB memory. The operating system is Windows 7. The deep learning framework is the Keras framework, and the programming language is Python 3.6.

We randomly scrambled the original images and its corresponding enhanced images and noise images. In this operation, the original images and the enhanced images and noise images remained corresponding. Then, 1000 original images and their corresponding 1000 enhanced images, 1000 Gaussian noise images, and 1000 salt and pepper noise images were selected randomly as the training set. The remaining images and their corresponding enhanced and noise images were used as the test set. After these steps, the training set and test set were randomly scrambled inside each to avoid continuous images. The above operations guaranteed that two images with the same fish distribution cannot appear in the training set and the test set. Finally, the training set contained 4000 images, the test set contained 2004 images, and 10% of the training set was used as the validation set. The Adam optimization algorithm was used for optimization. Table 1 shows the parameter settings. The linear rectified unit (ReLU) is the activation function in the convolution operation. Because the feature maps generated from the convolution of different columns need to be fused, the fusion operation requires the dimensions be the same except for the connection axis. Therefore, to ensure that the three obtained feature maps have the same size, the “same” padding method was used in the multi-column CNN, and the stride is set to 1.

### 3.1. Results of Fish Counting

**Figure 9:**
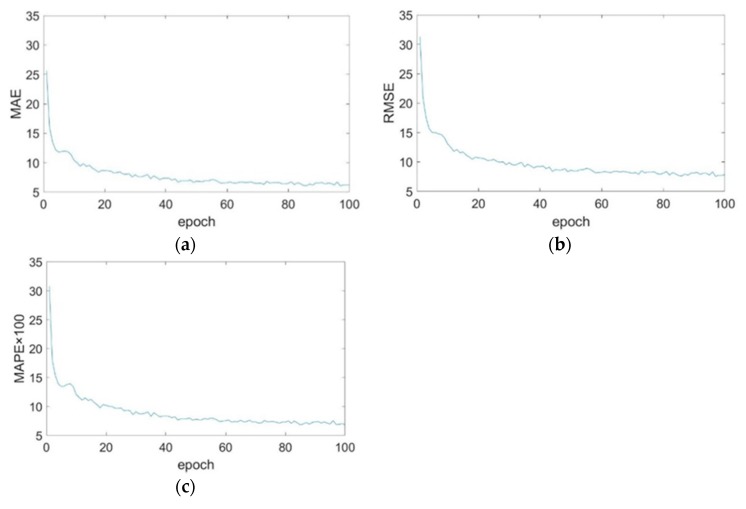
Mean absolute error (MAE), root mean square error (RMSE) and mean absolute percentage error (MAPE) change during epoch. (**a**) MAE; (**b**) RMSE and (**c**) MAPE.

Figure 9 shows the curves of MAE, RMSE, and MAPE during the validation process. The network weight keeps updating according to the change of MAE, RMSE, MAPE. The MAPE is small, and it is difficult to identify the changing trend; thus, the MAPE is multiplied by 100. When epoch reaches 20, the downward trend of the three curves starts to slow down, and curves basically dose not decline when epoch = 80. 

**Figure 10:**
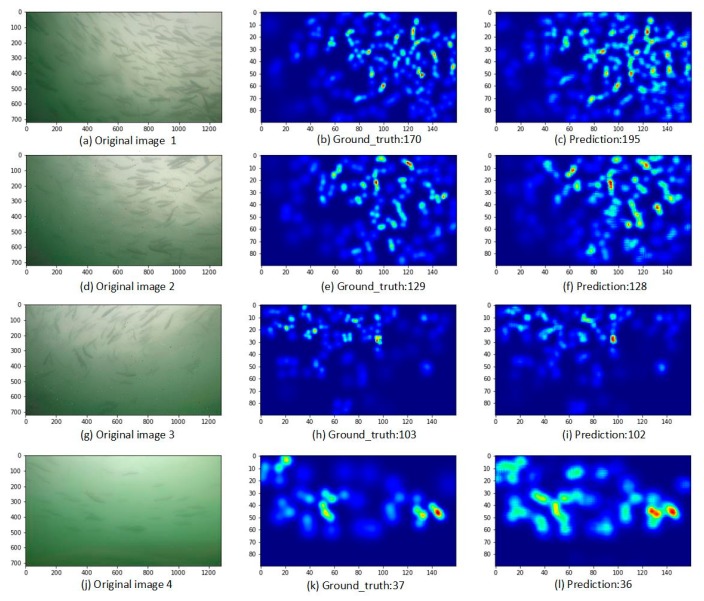
Fish counting results and density maps. (**a**,**d**,**g**,**j**) are original images; (**b**,**e**,**h**,**k**) are the ground truth density maps corresponding to (**a**,**d**,**g**,**j**) respectively; (**c**,**f**,**i**,**l**) are the estimated density maps corresponding to (**a**,**d**,**g**,**j**) respectively.

Finally, high-quality fish shoal density maps were generated using the trained model, as shown in Figure 10. Because the front end branches use three-times pooling operations, the length and width of the final generated density map become 1/8 those of the original, and the overall size is 1/64 that of the original. Figure 10 shows one group of relatively poor results (Figure 10a–c) and three groups of relatively good result (Figure 10d–l), one of which contains only a few objects (Figure 10j–l). Except for the counting results, the four groups of predicted density maps are in good agreement with the ground truth, which can overall reflect the distribution of fish shoal. 

**Figure 11:**
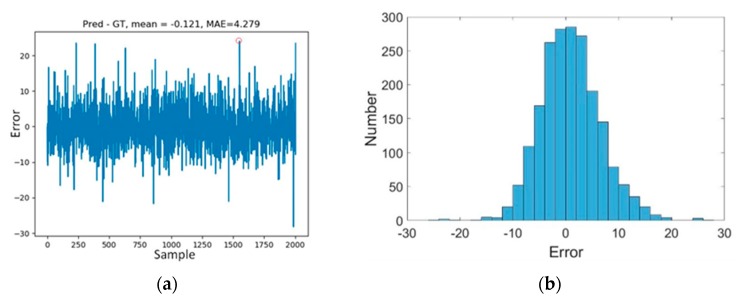
(**a**) Results of model test and (**b**) histogram of error between ground truth and the estimation.

Figure 11 shows the errors between the ground truth and the estimation of the model on all 2004 test images and the histogram of the error distribution. The errors are all within the range of −30 to 30, and most of the errors are stable in the range of -10 to 10. While also satisfying accuracy, the model can realize fish counting stably. The Pearson correlation coefficient between the estimation and the ground truth is 0.99. There are several large fluctuations in the test results. The reason for this may be label problems in the test data. To figure out this problem, we checked several samples with large errors. Take the sample with a red circled in Figure 11a as an example, the sample is the 1552nd sample in the test set, the error is 24, and it is found that there are fish are not labelled.

In order to compare the performance of the proposed model under different numerical ranges, we simply divided the dataset into five ranges (fewer, few, medium, many, large) according to the number of labels in the images, and calculated the number of samples and the accuracy within each range. Table 2 shows the counting results of the proposed model under different numerical ranges. It can be seen from Table 2 that the proposed model performs better with a large number of objects.

### 3.2. Discussion on Model Performance

#### 3.2.1. Dilated Convolution Neural Network

**Figure 12:**
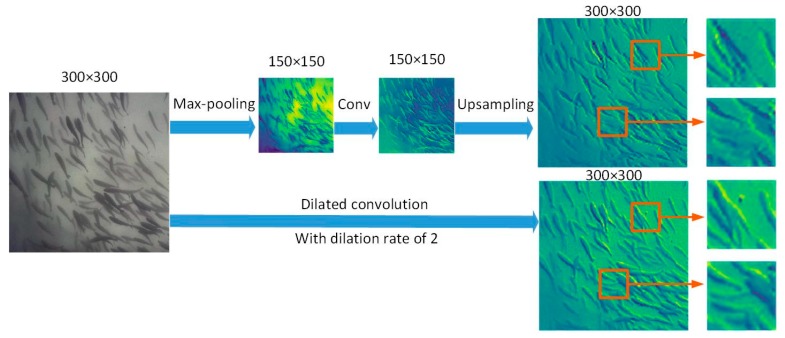
Comparison between dilated convolution and max-pooling, convolution, and upsampling.

In this study, dilated convolution [41] was used to retain more spatial structure information. Compared with the pooling+convolution+upsampling operation, dilated convolution has apparent advantages when keeping the size of the feature map unchanged. To further explain its principle, this article visualizes the process of extracting feature maps via convolution and dilated convolution. Taking Figure 12 as an example, the original input fish shoal image is processed by two different methods to generate a feature map of the same size. In the convolution method, the 2 × 2 max-pooling was used for downsampling, and then, a 3 × 3 convolution kernel was used to perform the convolution operation. Because the length and width of the generated feature map become 1/2 of those of the original images, upsampling must be used to restore the feature map to the original size. The dilated convolution method directly uses dilated convolution with a 3 × 3 kernel size and dilation rate of 2 to generate the same-sized feature map. There are 64 channels in both convolutions. Figure 12 shows that the feature image generated by the dilated convolution contains more detailed information (enlarged part). It shows that dilated convolution can be used as an alternative to a pooling-convolution structure, which simplifies the network structure and reduces the loss of more details.

#### 3.2.2. Comparison with Other Methods

To further compare the performance of the proposed model, experiments are carried out using CNNs and MCNN [21], respectively. CNNs are a branch of MCNN with the largest receptive field, and MCNN is the front end of the proposed model. Table 3 shows the results of three models, from which can be seen that the proposed model has achieved the best performance in four metrics, and the accuracy is up to 95.06%.

**Figure 13:**
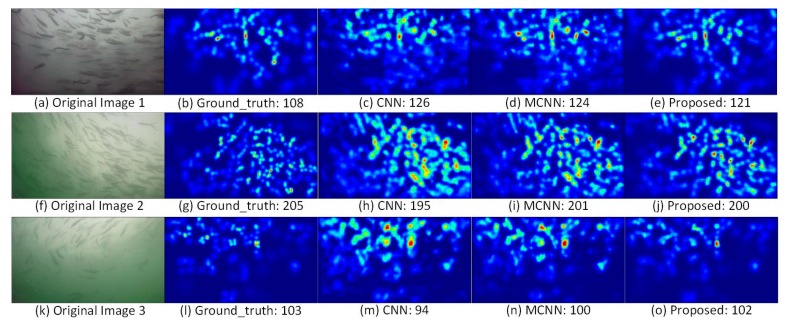
Test results of different models. (**a**,**f**,**k**) are original images; (**b**,**g**,**l**) are the ground truth density maps corresponding to (**a**,**f**,**k**) respectively; (**c**,**h**,**m**) are the estimated density maps generated by CNN corresponding to (**a**,**f**,**k**) respectively; (**d**,**i**,**n**) are the estimated density maps generated by MCNN corresponding to (**a**,**f**,**k**) respectively; (**e**,**j**,**o**) are estimated density maps generated by the proposed model corresponding to (**a**,**f**,**k**) respectively.

Figure 13 shows a comparison of the density maps generated by different models; Figure 13a,f,k are the original images; Figure 13b,g,l are the corresponding ground truth density maps; Figure 13c,h,m are the corresponding density maps generated by the CNN; Figure 13d,i,n are the corresponding density maps generated by the MCNN; Figure 13e,j,o are the corresponding density maps generated by the proposed model. Figure 13 shows that the density map generated by the proposed model has more detailed information and is most consistent with the ground truth. However, there are still problems with the density map. The image is relatively fuzzy, the distinction between individuals is not apparent; it is also difficult to observe more details. These are problems to be solved in the future.

**Figure 14:**
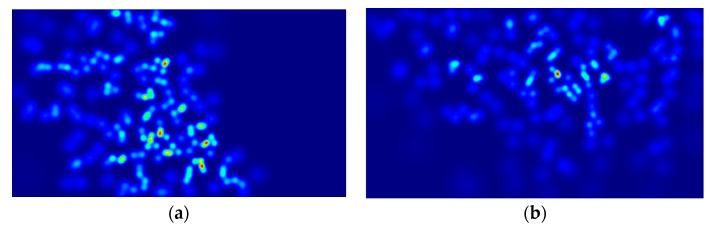
Comparison of the different distributions representing similar quantities: (**a**): 165 and (**b**): 162.

The density map can reflect the aggregation and dispersion of fish shoal. As shown in Figure 14, the numbers of fish in Figure 14a,b are 165 and 162, respectively. However, the distribution on the left side is more concentrated than that on the right side. In addition, when different-sized areas have the same number of fish, the smaller areas are denser; when there are different numbers of fish in the same-sized area, a larger number of fish indicates higher density. However, the fish in the water move in the three dimensions, and the accumulation and dispersion should also be in three-dimensional space. This two-dimensional spatial distribution cannot reflect the depth information of the fish shoal, which results in certain limitations on the reflection of the accumulation and dispersion of fish shoal. Only when the fish spread at a certain level (that is, the third dimension is limited), can a more accurate description of the fish gathering and scattering be obtained. Under certain special conditions, such as when fish colonies rise to the surface to feed and when the culture waters are shallow, the density map can effectively reflect the distribution of the fish shoal. In practical applications, the map can indirectly reflect starvation, abnormalities, and other states of the fish shoal according to the distribution of the fish shoal in the monitored area [42], thereby providing an important reference for production managers.

## 4. Conclusions

Underwater image processing technology is very challenging because of the complexity of the underwater environment and the enormous influence of lighting conditions. In this paper, a hybrid neural network model based on deep learning was proposed to generate a high-quality density map and to realize fish counting in underwater. The front end of the hybrid model uses CNNs with different convolution kernels to capture the feature information with different receptive fields. The back end of the model uses a deeper and wider dilated CNN to aggregate multi-scale context information. Through dilated convolution, the model can increase the receptive field without loss of resolution, thus improving the performance of the model. The accuracy of fish counting is 95.06%, and the Pearson correlation coefficient between the ground truth and the estimation is 0.99. The performance is better than that of CNNs and MCNNs. The results demonstrate the effectiveness of dilated convolution in reducing the loss of spatial structure information in the process of network transmission, which can be used to guide practical production. In addition, how to quantify the behavior state of fish shoal according to the density map will be addressed in future research.

## Figures and Tables

**Table 1:** Training parameter settings.

Parameter Name	Set Up	Parameter Name	Set Up
optimization algorithm	Adam	learning rate	1e-5
Gauss initialization	0.01 standard deviation	loss function	MSE in Keras
epoch	100	batch size	1 (online learning)
activation function	ReLU	padding	same

**Table 2:** Counting results of different numerical ranges.

Range Name	Range	Number	Accuracy
Fewer	<60	468	93.43%
Few	[60, 100)	656	94.21%
Medium	[100, 140)	376	95.77%
Many	[140, 180)	360	97.02%
Large	≥180	144	97.55%

**Table 3:** Comparison results of different methods.

Method	Metrics			
	MAE	RMSE	MAPE	Accuracy
CNN	8.85	11.37	10.39	89.61%
MCNN	7.85	10.10	8.82	91.18%
Proposed	4.29	5.57	4.94	95.06%
